# The Validation of the ‘CARe Burn Scale: Parent/Caregiver Form’—A Patient Reported Outcome Measure (PROM) Using Rasch Measurement Theory (RMT) to Assess Quality of Life for Parents or Caregivers Supporting a Child with a Burn Injury

**DOI:** 10.3390/ebj6020022

**Published:** 2025-05-07

**Authors:** Catrin Griffiths, Timothy Pickles, Ella Guest, Diana Harcourt

**Affiliations:** 1 Faculty of Medicine, Health and Life Science, Swansea University, Swansea SA2 8PP, UK; 2 Centre for Trials Research (CTR), Cardiff University, Cardiff CF14 4YS, UK; pickleste@cardiff.ac.uk; 3 Centre for Appearance Research (CAR), University of the West of England (UWE), Bristol BS16 1QY, UK; ella.guest@uwe.ac.uk (E.G.); diana2.harcourt@uwe.ac.uk (D.H.)

**Keywords:** burn injuries, scars, patient-reported outcome measures, PROMs, scar management, quality of life, well-being, coping, parents, caregivers

## Abstract

A PROM is a measure of patient needs and therapeutic progress. This paper outlines the validation of the CARe Burn Scale: Parent/Caregiver Form, a PROM that measures quality of life in parents/caregivers supporting a child with a burn injury. A literature review and interviews with sixteen parents and six burns health professionals informed the development of the PROM conceptual framework/draft form. Cognitive debriefing interviews with five parents and seven burns-specialist health professionals provided feedback to ascertain content validity, and two-hundred and four parents/caregivers took part in the field testing. Rasch measurement theory (RMT) analyses and internal consistency tests were conducted to create a shortened version and for psychometric validation. The final conceptual framework included eight domains/individual scales: *Physical Well-being, Confidence with Managing Burn Wound/Scar Treatments, Social Situations, Partner Relationship, Self-worth, Negative Mood, Parent Concerns about the Appearance of their Child’s Burn Wounds/Scars, and Positive Growth*. Seven scales had solutions from RMT analyses and passed internal consistency criteria. *Confidence with Managing Burn Wound/Scar Treatments* did not fit the Rasch model but was retained as a checklist based on theoretical insight. The CARe Burn Scale: Parent/Caregiver Form is the first and only burn-specific PROM that assesses parents’ own health needs when caring for a child with a burn.

## 1. Introduction

In the UK alone, 66,200 children and young people experienced a burn injury which required medical attention from 2013 to 2015 [1]. Children are at the greatest risk of experiencing a burn, as they physically develop and become increasingly active, impulsive and curious, but lack awareness [2,3]. Irrespective of a child’s age, a burn can have a significant psychosocial impact, both for the child and family members supporting them [4].

Adaptive parental coping is a key predictor of a child’s adjustment to a burn injury, with parents having a crucial role in supporting their child during rehabilitation and with managing the challenges of responding to other people’s reactions to their child’s scarring or altered appearance [5,6]. However, burn injuries place significant pressure on parents, which can affect their emotional well-being and ability to cope [7,8]. Many are responsible for their child’s wound and scar management, including daily dressing changes, pressure garment application, creaming, massage, washing and dressing, and trying to avoid infections [9,10]. Particularly in the early weeks post injury, these experiences are often painful and distressing for children, and stressful for parents to witness [11]. Parents can face financial and marital challenges when taking care of their injured child, alongside other responsibilities such as domestic chores, work, and looking after uninjured siblings [9,12,13,14,15]. Although many parents cope well with these pressures, others report a detrimental impact on their well-being [16].

Parents are often concerned about the appearance of their child’s scars and can experience guilt, anxiety, depression, and post-traumatic stress symptoms (PTSS). For example, 43–69% of parents report anxiety [17,18] and 19–44% report depression during the acute stage of burn treatment [18,19]. PTSS are also reported by 42% of mothers at 1–2 years after the burn injury, with 19% still experiencing PTSS 10 years post injury [20]. Chronic parental PTSS are also associated with the development of child PTSS [12,21]. These emotional difficulties are unpleasant and challenging for parents and they can also impact parents’ ability to look after their child.

Parental guilt or internalised blame after a burn injury is very common [22,23,24]. A total of 27–81% of parents report feeling guilt related to their child’s burn injury [5,7,20], irrespective of whether they were present at the time of the burn injury or not [15,24]. Parents can also encounter enacted blame from family members, partners, or health professionals in relation to the event that injured their child, or about their child’s adherence to treatment [23]. It is vital for clinicians to help parents cope with these feelings, since unmanaged parental guilt can impact parents’ emotional well-being, their confidence in parenting, and their ability to cope more generally [9,23,25].

Research into the psychosocial impact of supporting a child with a burn has indicated that the severity/size of a child’s burn does not reliably predict parental distress [16,18,26,27]. All parents, regardless of the severity of their child’s injury, are at risk of experiencing psychological difficulties. Some risk factors that have been found to increase the likelihood of parent distress include parents having a lower emotional stability [18], family conflict [4], poor family functioning [18], and being a mother (rather than a father) [12]. This suggests that parental coping skills and access to family social support are better predictors of parental coping and distress than burn size/severity. It is therefore essential that health professionals identify parents’ needs to ensure that they receive the best possible support and so that parents are equipped with the psychological and practical resources that they need to effectively support their child and family, as well supporting their own psychological well-being.

Health professionals often use patient-reported outcome measures (PROMs) (i.e., psychometrically validated health questionnaires) to identify the health needs and therapeutic progress of patients and caregivers. Accordingly, the UK’s National Health Service (NHS) Next Stage Review recommended the use of PROMs to identify patient needs [28]. However, a national review of burn care in the UK concluded that PROMs were not used consistently and that there was a lack of burn-specific outcome measures designed to assess the needs of those affected [29]. Likewise, qualitative research with burn care psychosocial specialists highlighted a lack of available burn-specific PROMs for patients and their families [30].

There are two existing burn-specific PROMs, which include some items that measure some aspects of parents’ health and well-being when supporting a child with a burn. The Brisbane Scar Profile has two Parent/Caregiver Forms [31], which include a couple of subscales that measure ‘Parent and Family Concerns’ and ‘Parent Worry’ for parents/carers of children aged less than 8 years, or 8–18 years. Likewise, the Children’s Burns Outcome Questionnaire for ages 5–18: Parent-report Form [32] has two sets of items which assess the effect of their child’s health or behaviour on the parent’s own life (such as their domestic and social life and work) and their worry or concern about their child’s health and recovery after a burn. However, these PROMs only include a few subscales, or a few items related to parental well-being, which are within PROMs where the key focus is measuring the child’s health after a burn. Notably, no PROM currently exists that is specifically designed to measure parents’ needs and quality of life when supporting a child with a burn.

This paper outlines the validation of the CARe Burn Scale: Parent/Caregiver Form, a burn-specific PROM that assesses health outcomes for parents/caregivers supporting a child with a burn. This is a scale for any adult with parental/caring responsibility for a child who has had a burn. From here on in this paper, for brevity, the word ‘parent’ will be used to mean any parent/caregiver.

## 2. Methods

### 2.1. Background Scale Development Process

The CARe Burn Scale: Parent/Caregiver Form was developed following an established three-stage development and validation process, which has been outlined by Cano et al. (2004) and the Medical Outcomes Trust and is considered the gold standard for developing and evaluating PROMs [33,34,35,36]. This involved item generation (developing a conceptual framework using a literature review, qualitative interviews with parents, and expert opinion), item reduction, and psychometric evaluation.

#### 2.1.1. *Item Generation*

Semi-structured interviews were conducted either in person or over the telephone (depending on each participant’s preference) by the first author (an expert in burns and qualitative research) with sixteen parents (twelve female) of children who had experienced a burn, and six burn-specialist health professionals (one psychotherapist and five clinical psychologists) to explore, in-depth, the impact of a child’s burn on both the child and their parents.

**Figure 1:**
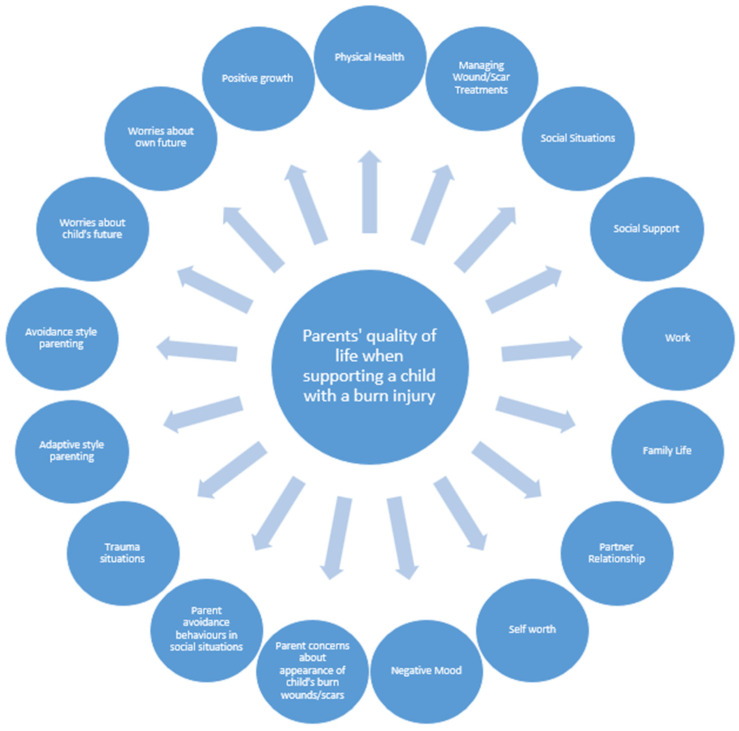
Draft conceptual framework before psychometric field testing.

Interview questions explored parents’ experiences of supporting a child with a burn, including how the burn injury had affected their child’s health, in what ways parents helped support their child, how supporting their child with a burn had affected the parent’s own health, well-being, and relationships with others, what skills/ways of coping had helped them, and any support needs they had. Interviews were recorded, transcribed verbatim, and subjected to a thematic analysis [37]. A draft conceptual framework was then developed from these findings, which outlined the key themes/domains reflecting parents’ quality of life and well-being when supporting a child with a burn (see Figure 1 for draft conceptual framework).

Based on the parent interview data, an extensive list of a total of 197 items was created to develop a draft version of the measure, covering all domains in the conceptual framework, using parents’ own words or phrases to increase the content validity of the items.

A review was conducted of existing parent-reported PROMs used in paediatric burn care research to measure parents’ quality of life and coping. PUBMED and MEDLINE research databases were used to find relevant research using the following main search terms: “PROM” OR “patient reported outcome measure” OR “outcome measure” AND “well-being” OR health OR “quality of life” OR coping AND parent OR caregiver OR mother OR father AND Burn OR “burn injury”. No burn-specific parent-reported outcome measures that specifically measured parental health and quality of life were identified. Measures of parental coping more generally, rather than burn-specific coping, were obtained through this search, reviewed, and any topics included in those scales that were not discussed in the interviews were added to the draft measure.

Cognitive debriefing interviews [33,36] were conducted with five parents who had taken part in the qualitative interviews who reviewed the draft scale and gave feedback on their understanding of the items, the response categories, and measure instructions, to ensure that the scale had good face/content validity and was relevant and understandable to parents of children with burn injuries [38]. A think aloud approach was used, which is a common technique for cognitive debriefing interviews when developing PROMs [39]. These involved participants being sent the draft version of the measure ahead of the interview, which took place over the telephone. The interviewer (the first author, who is an expert in qualitative analysis) went through each scale item in turn, asking the participant to comment on their understanding of the meaning of the item, its relevance, and readability, the related response categories, and the instructions for the measure. Participants also made suggestions for improving the measure in general. Minor changes to the wording of questions (i.e., using simpler language or using more burn-specific examples) were made in line with participants’ feedback.

Seven burn-specialist multidisciplinary health professionals (four clinical psychologists, two nurses, and one psychotherapist) from UK burn services provided feedback on the draft Parent/Caregiver CARe Burn Scale to ensure it was comprehensive. These burns specialists also showed the draft scale to their multidisciplinary teams (which included nurses, play therapists, occupational therapists, physiotherapists, and surgeons) and then provided feedback on behalf of themselves and their team. In response to health professional feedback to maximise the practicality of the scale, all items within an individual subscale were phrased either positively or negatively to reduce the need for rescoring specific items.

#### 2.1.2. Field Testing for Item Reduction and Psychometric Evaluation and Validation

The final draft scale used in field testing had 197 items covering 17 domains/individual scales:

#### 2.1.3. Description of Individual Scales

1. *Physical Health*: measured parents’ physical health and their physical abilities.
2. *Confidence with Managing Burn Wound/Scar Treatments*: the extent to which parents feel confident with supporting their child during a range of different wound/scar treatments such as washing, dressing, physiotherapy exercises, and dressing or bandage changes.
3. *Social Situations*: the extent to which parents feel confident during challenging social situations in which other people may look, touch, or ask questions about their child’s burn wounds/scarring.
4. *Social Support*: measured parents’ perceptions of the quality of the social support available to them from friends, family, and health professionals.
5. *Work Life*: measured parents’ perceptions of the quality of their work life.
6. *Family Life*: measured parents’ perceptions of the quality of their family life and relationships.
7. *Partner Relationship*: measured parents’ perceptions of the quality of their partner relationship whilst supporting a child with a burn injury.
8. *Self-worth*: the extent to which a parent has positive feelings about themselves.
9. *Negative Mood*: the extent to which a parent reports low/negative mood.
10. *Parent Concerns about the Appearance of their Child’s Burn Wounds/Scars*: how bothered parents are about the appearance of their child’s burn wound/scarring.
11. *Parent Avoidance Behaviours in Social Situations*: the extent to which parents avoid looking at and touching their child’s burn wound/scars and avoid public places and discussing their child’s wound/scars with others.
12. *Trauma Symptoms*: the extent to which parents experience negative psychological and behavioural symptoms such as negative thoughts, flashbacks, bad dreams, and anxiety related to the event that injured their child, or the events/treatment that happened afterwards.
13. *Adaptive Coping Parenting*: the extent to which a parent engages in ways of coping and supporting their child that are adaptive and proactive.
14. *Avoidance Coping Parenting*: the extent to which parents find it difficult and avoid challenging situations and making decisions when supporting their child after their burn injury.
15. *Worries about their Child’s Future*: the extent to which parents are concerned about how their child’s burn injury will impact their future in terms of their appearance, physical and psychological health, romantic relationships, and work.
16. *Worries about their own Future*: the extent to which parents are concerned about how their child’s burn injury will impact their own future in terms of their physical and psychological health, partner relationship, and career.
17. *Positive Growth*: the extent to which parents report positive outcomes or personal development after supporting a child with a burn injury.

Most of the individual scales (i.e., *Physical Health, Confidence with Managing Burn Wound/Scar Treatments, Social Situations, Social Support, Work, Family Life, Partner Relationship, Self-worth, Negative Mood, Parent Avoidance Behaviours in Social Situations, Trauma Symptoms, Adaptive Coping Parenting, Avoidance Coping Parenting, Worries about their Child’s Future, Worries about their own Future*) used a frequency response category (i.e., never, sometimes, often, most of the time, always).

The *Parent Concerns about the Appearance of their Child’s Burn Wounds/Scars* scale used an extent response category (i.e., not at all, a little, somewhat, quite a bit, a lot) and the *Positive Growth* scale used an agreement response category (i.e., definitely disagree, somewhat disagree, somewhat agree, definitely agree).

The draft Parent/Caregiver CARe Burn Scale (which included a set of demographic questions) was field-tested in 11 NHS Burn Services across the UK with parents of children aged 18 and under at time of their burn injury. The children/young people that parents/caregivers were supporting could have had a burn injury of any size or location on the body. They needed to have a sufficient comprehension of English to complete the questionnaire. Burns health professionals (i.e., psychologists, research administrators, nurses) identified potential participants either at burn clinics or via their patient database. Participants were informed that the study was testing a questionnaire that measured the health and well-being of parents/caregivers who were supporting a child with a burn. Eligible participants were handed paper questionnaires (at clinics) or mailed out questionnaires (patients identified on patient database). Participants could complete the questionnaire on paper or online via a web survey link (www.qualtrics.com).

Approvals were obtained from the University of the West of England Faculty of Applied Science Research Ethics Committee and an NHS research ethics committee (NHS IRAS number: 167766). Participants provided written/online informed consent before completing the questionnaire.

### 2.2. Data Analysis

#### 2.2.1. *Rasch Measurement Theory Analyses*

The Rasch measurement model and Rasch measurement theory (RMT) analyses [40,41,42] were used for item reduction using RUMM2030 [43]. International health measurement instrument experts COSMIN state RMT is a suitable method for this purpose, along with Item Response Theory (IRT) and classical test theory (CTT) [44]. RMT was used for item reduction for all the previous scales developed as part of the CARe Burn Scales portfolio and, for consistency, was therefore chosen for item reduction for this scale [45,46].

Each individual scale measuring the domains outlined in the conceptual framework was analysed using the following criteria:

#### 2.2.2. *Item Fit Statistics*

RMT analysis investigates whether the study data are consistent with the responses predicted by the Rasch measurement model. Two indicators were investigated: (1) item–trait interaction (a non-significant (*p*-value > 0.05) chi-square value indicated negligible deviation between observed data and expectations of the model); (2) the residual for each item in the range of −2.5 to +2.5 indicated a good fit [47], and should also have non-significant chi-square and F-statistic values (Bonferroni adjusted α = 0.05).

#### 2.2.3. *Person Separation Index (PSI)*

Internal consistency within RMT was assessed through the calculation of the Person Separation Index (PSI). The criterion for evidence of internal consistency, much like that of a Cronbach’s α, was PSI ≥ 0.7 [44].

#### 2.2.4. *Local Dependency*

For each pair of items within a scale, a residual correlation >0.2 above the mean residual correlation (of all item pairs for that scale) indicates a problem with the fit, suggesting the existence of unexpected associations within the set of items [48].

#### 2.2.5. *Unidimensionality*

Smith’s procedure based on paired *t*-tests was used to check for unidimensionality to identify if the person estimates derived from the most diverse subsets of items were significantly different [49]. If the proportion, or the lower bound of the 95% confidence interval, of significant (*p* < 0.05) *t*-tests was less than 5%, it confirms unidimensionality.

#### 2.2.6. *Differential Item Functioning*

Different Item Functioning analysis (DIF) was conducted to assess the extent to which item parameters remain invariant across different groups of patients [50]. Item difficulties were compared across the following: child’s gender (male vs. female), child’s current injury status reported by parent/carer (burn wound vs. burn scar only vs. no wound/scar) and child’s body part affected (hands, bottom, upper legs, lower legs, feet vs. other areas). This checked for the possible bias that might result in a misfit of the data to the model. Uniform and non-uniform DIF were investigated graphically (inspection of item characteristic curves (ICCs) for different groups) and by the results of the analysis of variance Bonferroni-adjusted α = 0.05.

#### 2.2.7. *Targeting and Item Locations*

Distributions of item and person locations were graphically compared to identify whether they covered more or less of the same areas of the Rasch continuum. Large (15%) floor and ceiling effects suggest a targeting problem.

#### 2.2.8. *Item Thresholds*

The response category structure was assessed to determine whether the item response format was operational. Item thresholds, which are the transition points between response categories, should be ordered in a logical, progressive manner. If this was not the case, then the item was considered to have disordered thresholds.

#### 2.2.9. *Internal Consistency*

Traditional psychometric analyses via classical test theory (CTT) were conducted on the data to investigate how the scale operated based on CTT criteria: Cronbach’s alpha (for each individual scale) and item-total correlations were calculated to assess internal consistency. The criterion for evidence of internal consistency was α ≥ 0.7 [44]. Analyses were undertaken using IBM SPSS Statistics 23 [51].

## 3. Results

### 3.1. Participants

A total of 204 participants completed the CARe Burn Scale: Parent/Caregiver Form. Participant characteristics are shown in Table 1. The largely supported rule of thumb is that in order to perform accurate and precise RMT analyses with item calibrations within ±0.5 logits, the advised sample size is 250 [52]. Our sample is slightly less than 250, but it is still valid to perform RMT analyses on a sample of this size.

Out of the two-hundred and four participants, one parent had a child who had just turned the age of 18 (but who was injured when they were under the age of 18). Given the short timeframe between turning 18 and as the majority of their experiences related to supporting a child who was under the age of 18 at time of injury, the team agreed to include this parent in the analysis as the impact on the parent’s well-being would not be significantly different.

### 3.2. Item Reduction and Scale Formation

Of the seventeen scales tested, a solution was found via RMT analyses for seven (Table 2): Physical Health, Social Situations, Partner Relationship, Self-worth, Negative Mood, Parent Concerns about the Appearance of their Child’s Burn Wounds/Scars, and Positive Growth. Of the ten individual scales for which a solution was not found via RMT analyses, nine scales were removed, and one, Confidence with Managing Burn Wound/Scar Treatments, was retained as a checklist based on health professionals’ feedback that this was clinically important. Of the seven scales where a solution was identified via RMT analyses, 89 items across all seven scales were reduced to 33 (see Table 2 for the items in each individual scale). RMT analyses showed that there was no solution to obtain an aggregate/total score to combine all of the individual scales. Therefore, each individual scale was scored independently.

Scale internal consistency was generally supported by a high PSI, though this was marginally low for *Partner Relationship*. The fit to the Rasch measurement model was good, with all item–trait interactions being non-significant and no items with fit residuals out of the range or presenting significant Χ2 values. For *Physical Health* and *Partner Relationship*, one item in each of these scales exhibited marginal uniform DIF by body part affected. The vast majority of items did not exhibit DIF, suggesting that items remain invariant across different groups of patients.

All final scale solutions contain no items with reversed thresholds. However, five of the seven solutions required response categories to be collapsed for this to be the case. For *Physical Health*, the second and third categories were collapsed for a single item only, and for *Social Situations*, *Partner Relationship*, and *Negative Mood*, the third and fourth categories were collapsed (though only for two items in the case of Partner Relationship). All pairs of items within each scale had a residual correlation less than 0.2 above the mean residual correlation (of all item pairs for that scale), supporting local independence amongst items. Unidimensionality was confirmed via Smith’s procedure for all seven scale solutions [49]. Item locations were well spread out, indicating that the scale defined a continuum. *Physical Health* and *Partner Relationship* had items with DIF issues. However, the evidence for these DIF issues is weak (*p*-value just less than the α = 0.05 Bonferroni-corrected level) but are reported for full disclosure.

### 3.3. Internal Consistency

All scales with solutions via RMT analyses passed criteria for internal consistency (Table 2): Cronbach’s alpha > 0.81 and item-total correlation coefficients > 0.59.

### 3.4. Checklist

A Rasch solution was not found for the *Confidence with Managing Burn Wound/Scar Treatments* scale; however, it was retained (with all original items) as a checklist based on theoretical insight. This was based on the feedback from the clinicians involved in the pretesting stages of the development of this scale, who reported that this scale would still be useful for their clinical practice with patients. For all items, ‘A lot’ or ‘N/A’ were the most commonly endorsed categories (Table 3). This checklist is scored by summing all items within the scale. Clinicians and researchers can use this scale to collect further information about this construct; however, this checklist should not be used for psychometric analysis, since it is not psychometrically valid.

**Figure 2:**
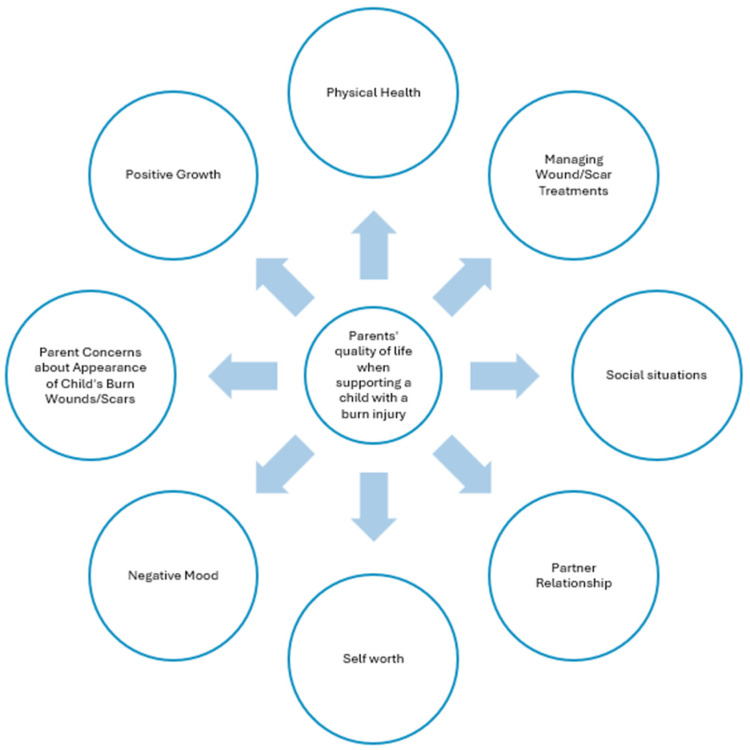
Final conceptual framework.

See Figure 2 for the final conceptual framework for the Parent Form after the field testing study.

## 4. Discussion

The CARe Burn Scale: Parent/Caregiver Form is the first and only validated burn-specific PROM designed to specifically measure parents’ own health and support needs when caring for a child with a burn. This PROM was developed in line with the international guidelines for the development and validation of health outcome measures outlined by the Scientific Advisory Committee of the Medical Outcomes Trust reported by Cano et al. [35]. As per these guidelines, parents themselves had an integral role in the development of this new PROM. Their interview data informed item generation and parents reviewed and provided feedback on draft versions of the scale. The PROM therefore reflects the key experiences that parents report as important to their health when supporting a child with a burn. It is vital that clinicians have access to and use a burn-specific PROM to identify parents’ needs to provide the best possible support. Ensuring that parents are well supported during this traumatic time can improve their coping skills and well-being, which is associated with better adjustment and psychological outcomes for their children [53].

The results from this study showed that RMT analyses identified seven unidimensional scales which measured parents’ health: Physical Well-being, Social Situations, Partner Relationship, Self-worth, Negative Mood, Parent Concerns about the Appearance of their Child’s Burn Wounds/Scars, and Positive Growth. A solution was not found for Confidence with Managing Burn Wound/Scar Treatments, although it was retained as a checklist based on theoretical insight from clinicians.

For the seven scales where a solution was found via RMT analyses, the total items were reduced to 33. The Cronbach’s alphas for each scale were all over 0.81, reflecting strong internal consistency, and only two out of thirty-three items exhibited DIF, suggesting that the items exhibit measurement invariance across the different demographic parent groups.

The individual scales in the Parent Form cover a wide range of physical, psychological, and social experiences that parents themselves reported as key to their health when supporting a child with a burn. The *Physical Well-being* scale measures parents’ physical health. In the qualitative interviews that informed the PROM item generation, many parents reported finding it difficult to engage in healthy behaviours such as exercising and eating healthily due to a lack of time or energy and reported that this affected their physical health. Research supports this potential impact on physical health, with one study finding that parents reported more cardiovascular health problems every year for 4 years after their child was injured compared to baseline [54]. In addition, parents of children with a burn injury and spouses of burn patients have been found to display a supressed immune response 72 h after their child’s or partner’s hospital admission, although this function improved 2 to 5 weeks later, suggesting only short-term effects on immune functions [55].

The *Social Situations* scale measures parents’ confidence when responding to unwanted attention or questions about the appearance of their child’s burn wounds/scars and their confidence about discussing the event that caused their child’s burn. For example, children with an altered appearance, such as those with burn wounds/scars, are at risk of receiving unwanted attention such as staring, teasing, or questions about their injury. Without adequate skills to respond to these challenging social encounters, children can experience social anxiety and are at risk of developing behavioural difficulties [56,57,58,59,60]. According to Social Learning Theory, children learn social skills from significant others such as parents and siblings, who are also a key source of social support for burn patients [61,62,63]. Measuring parents’ own confidence in responding to this unwanted attention is key to ensuring that health professionals can identify when to intervene and offer appropriate support. When parents are confident at managing these challenging social situations, they can then model these skills to their children and, with practice, children can become more confident when navigating these difficult encounters [64].

The *Partner Relationship* scale measures the quality of the parental relationship when they are supporting their child with a burn. Research indicates that when a child suffers a burn, it can impact the parental relationship, resulting in a greater likelihood of partner conflict and higher divorce rates compared to normative populations [65,66]. Parents who have an adaptive relationship with their partner, where they are able to openly discuss issues and support each other, are more likely to be resilient to the potential stress of supporting a child with a burn [53,67]. It is vital for parents to have the opportunity to express any support needs they might have in terms of their relationship with their partner, so clinicians can support both parents to help them care for each other and their child. This is the first burn-specific PROM that measures the quality of the partner relationship.

The *Self-worth* and *Negative Mood* scales measure positive feelings about oneself and negative affect, respectively. A substantial body of research suggests that supporting a child with a burn can have a significant impact on parents’ mental health and mood, with 15–44% of parents reporting depression and 23–69% reporting anxiety symptoms [5,18,19,26]. Despite this, parents also describe positive emotions such as relief and gratitude and report focusing on positive experiences associated with supporting their child, such as watching their child do well during recovery and gratitude for the support from staff, friends, and family [68].

The *Parent Concerns about the Appearance of their Child’s Burn Wounds/Scars* scale measures parents’ opinions of how their child’s wounds or scars look and includes the location, size, shape, and overall appearance of their child’s wound or scars. A parent’s own perception of their child’s burn scars is a key factor, which influences children’s own evaluation of their scarring and adjustment [69]. It is therefore vital to capture parents’ own opinions of their child’s scarring, so that clinicians can help parents to manage any concerns they might have.

The *Positive Growth* scale measures positive experiences that parents report after supporting a child with a burn. Tedeschi and Calhoun [70] defined post traumatic growth (PTG) as positive life changes after experiencing trauma. Often, people re-evaluate their lives and personal priorities when faced with their own or their loved one’s mortality. This can result in closer relationships with loved ones, becoming more accepting of those less fortunate, and feeling stronger or more confident as a person [70]. Overall, there is a lack of research investigating post traumatic growth amongst parents of children with burns. Research by Gavrilova et al. (2024) found that around half of parents/caregivers in their study reported moderate/high levels of post traumatic growth after their child experienced a burn [71]. Similar findings were reported in recent research by Zhao and colleagues in China [72], with 74% of parents/caregivers reporting moderate levels of post traumatic growth. Results that were particularly noteworthy were related to the trajectory of PTG over time, with PTG increasing at 0–6 months post burn, then decreasing at 6–24 months post burn, and PTG then increasing again at 24 months post burn. These findings highlight the importance of measuring the potential positive impact that a burn might have on parents, and also the importance of collecting regular outcome measurement data at long-term follow ups to better understand parents’ longer-term support needs. The creation of the *Positive Growth* scale in this Parent Form allows researchers and clinicians in the future to explore the possible post traumatic growth effects for parents when caring for a child with a burn.

The *Confidence with Managing Burn Wound/Scar Treatments* scale measures parental confidence when they manage their child’s wound/scar treatments, such as dressing/bandage changing, creaming/massage, washing and dressing, and managing pain and itching. Parents often find scar management challenging due to their child’s discomfort and attempts to avoid treatments [52]. Many parents report finding it difficult to see their child in pain [73] and some find it hard to look or touch the injured area because doing so brings up feelings of blame and guilt related to the accident. Yet, adherence to these techniques is a key predictor of wound healing and longer-term scarring outcomes [74,75]. It is therefore vital to ensure that parents’ experiences and confidence at conducting wound/scar management treatments are measured, so that those parents that are struggling can be identified and supported.

### 4.1. Strengths

A key strength of the CARe Burn Scale: Parent/Caregiver Form is that it is the first and only existing validated burn-specific PROM to identify parents’ own health and support needs when caring for a child with a burn. Using this scale in clinical practice can help clinicians to better identify parents who might need extra support when caring for their child.

Another strength of this research was the large number of different NHS Burn Services across the UK, including services in England, Scotland, and Wales, that took part in recruitment. The data collected therefore represent patients nation-wide, which increases the representativeness of the data.

The CARe Burn Scale version described in this paper is only valid for measuring parents’ own health when supporting a child with a burn. However, it is part of the CARe Burn Scales portfolio, which is a set of age-appropriate burn-specific PROM forms developed by the authors that assess quality of life and health outcomes for all ages of burn patients and their caregivers. The portfolio includes the Child Form (for children aged 0–8 years), the Young Person Form (for young people aged 8–17 years), the Adult Form (for adults aged 18 and over), and the Parent/Caregiver Form [45,46]. Each CARe Burn Scale Form was developed in collaboration with burn patients, family members, and health professionals, using burn patient/family member and health professional interview data, and went through rigorous psychometric testing, which showed evidence of reliability, validity, and responsiveness [30,76,77,78,79]. The CARe Burn Scales have also been translated and validated in other languages, including Finnish and Norwegian [80,81].

### 4.2. Limitations

The sample was relatively homogeneous in terms of gender (83.3% female) and ethnicity (73.5% White). Sexual orientation and biological sex data were not collected as part of this field-testing study, or in the previous interview study, which informed the item generation for this scale. Likewise, all participants were English speakers. This limits the generalisability of the results. Future research could test the CARe Burn Scale: Parent/Caregiver Form with more gender and ethnically diverse samples, as well as with parents with limited English proficiency. Translation and testing of the Parent Form into other languages is needed.

We did not undertake exploratory factor analyses to explore the possibility of item cross-loading across domains. Items were generated in such a way that we believe that items were in the correct conceptual domains. Other aspects of psychometric evidence such as convergent and concurrent validity were not tested in this study, as it was beyond the aims of this research. However, the authors have collected these data as part of another study and are currently writing up further research to show evidence of the convergent and concurrent validity of the CARe Burn Scale: Parent/Caregiver Form.

### 4.3. Future Recommendations

A further large-scale study, testing the responsiveness of the Parent/Caregiver Form with 320 parents, has been conducted since this current study [76]. The results showed that the Parent/Caregiver Form was responsive and able to detect change over three timepoints up to 6 months post burn (i.e., 4 weeks, 3 months, and 6 months after their child’s burn injury). Based on these findings, it is recommended that data from the Parent/Caregiver Form is collected at these timepoints to provide researchers/clinicians with the optimum chance of identifying clinical changes in parents’ quality of life.

Minimal Important Difference (MID) values for the Parent Form were also calculated and are presented in the related paper [76]. A MID value is the change (the difference in scores between two timepoints on an individual scale) that scores must change by to show a ‘small but important change’ in that measure. MID values are vital when using PROMs in clinical care, as they can be used by clinicians and researchers who are using the Parent/Caregiver Form to identify whether the quality of life of parents they are working with has meaningfully changed between two timepoints. The MID values for the Parent/Caregiver Form are outlined in a table in a previous paper [76]. It is recommended that researchers/health professionals use the MID values when collecting data from the Parent/Caregiver Form over more than one timepoint, to identify whether parents have meaningfully changed in the domains outlined in the Parent/Caregiver Form. Health professionals who wish to do this need to collect parent data using the Parent/Caregiver Form, score them using the scoring templates (freely accessible via www.careburnscales.org.uk, (accessed on 28 January 2025), identify the MID values [76], and then compare subscale scores between the two timepoints. If the absolute difference between the two time periods is greater than or equal to the MID value, it can be ascertained that that person has meaningfully changed on that subscale (improved or deteriorated, depending on whether scores have increased or decreased in the follow-up timepoint).

The CARe Burn Scales (Child, Young Person, Parent, and Adult Forms) are now the recommended PROMs for all NHS Burn Services to use as outlined in the 2024 British Burns Association National Outcome Measures in Adult and Paediatric Burn Care guidelines [82].

## 5. Conclusions

Parent/caregiver coping is a key predictor of child well-being and adjustment to a burn [83,84,85]. It is vital that validated burn-specific patient-reported outcome measures (PROMs) are available for health professionals to use in their practice. This will ensure that parents’ and other carers’ needs are captured so that relevant support can be offered, which reduces the likelihood of their own mental health suffering and its related impact on their child’s well-being. The CARe Burn Scale: Parent/Caregiver Form is the first PROM developed and validated to identify parents’ and other caregivers’ own health needs when supporting a child with a burn.

The CARe Burn Scale: Parent/Caregiver Form is now available for clinical/research use to identify the health needs of parents supporting a child with a burn (see www.careburnscales.org.uk (accessed on 28 January 2025) to access the full set of CARe Burn Scales).

## Figures and Tables

**Table 1:** Participant characteristics (n = 204).

	Demographics	N	%
Parent Age	Mean 36.5 (SD 7.30), range 20 to 63	182	
Parent Gender	Male	14	6.9
	Female	170	83.3
Parent Marital Status	Married	125	61.3
	Civil Partnership	5	2.5
	Single, never married	12	5.9
	Separated	3	1.5
	Divorced	12	5.9
	Cohabiting	25	12.3
	In a relationship but not living together	2	1.0
Parent Ethnicity	White British	150	73.5
	White Other	13	6.4
	Asian or Asian British: Indian	5	2.5
	Asian or Asian British: Pakistani	4	2.0
	Asian or Asian British: Other	1	0.5
	Black or Black British: Black African	3	1.5
	Chinese or Other Ethnic Group: Chinese	2	1.0
	Mixed: White and Black Caribbean	3	1.5
	Mixed: Other Mixed	2	1.0
	Other	1	0.5
	Rather not say	2	1.0
Child Current Age	Mean 4.7 (SD 4.13), range 0 to 18	186	
Child Age at Injury	Mean 3.3 (SD 3.84), range 0 to 16	184	
Time Since Injury (Years)	Mean 1.4 (SD 2.02), range 0 to 17	182	
Child Gender	Male	104	51.0
	Female	80	39.2
Child Injury Status	Burn wound	7	3.4
	Burn scar	99	48.5
	Both wound and scar	5	2.5
	No wound scar	71	34.8
Child’s Body Part Affected	Head or face	47	23.0
	Neck	35	17.2
	Chest	60	29.4
	Abdomen	26	12.7
	Back	19	9.3
	Lower arms	36	17.6
	Upper arms	49	24.0
	Hands	70	34.3
	Bottom	5	2.5
	Upper legs	33	16.2
	Lower legs	20	9.8
	Feet	25	12.3
Cause of burn	Flame	11	5.4
	Liquid	110	53.9
	Contact	46	22.5
	Electricity	2	1.0
	Chemical/acid	1	0.5
	Other	16	7.8

Percentages in the above table may not sum to 100% or to 204 participants due to missing data and as they show the share of given group in the whole sample of 204 parents of burn patients.

**Table 2:** Summary of CARe Burn Scale—Parent Coping Form Psychometric Analyses.

	Rasch Analyses												Traditional Psychometric Analyses	
CARe Burn Scale	Number of Items Retained in Final Version	Item-Trait Interaction X^2^; df; *p*	Person Separation Index	Number of Items with Fit Statistics Residuals Outside the Band of −2.5, +2.5	Items with Significant Χ^2^ Value *	Items with Significant F-Statistic Value *	Number of Items with Thresholds Reversed	Number of Pairs of Items with Residual Correlation > 0.2 Above the Average	Number of Items with DIF ****	Unidimensionality Tests: Highest Eigenvalue ** (Proportion of Significant *t*-Tests ***)	Item Locations Logits (Range of Items) [Range of Category Thresholds]	Person Location Logits (Mean, SD) and Person Fit Residual [mean, SD]	Cronbach’s Alpha	Item-Total Correlation Mean (Range)
Physical health	3	6.17; 6; 0.404	0.827	0	0	0	0	0	1	1.634 (3%)	(−0.415, 0.395) [−4.330, 4.331]	(1.455, 2.597) [−0.510, 0.948]	0.876	0.778 (0.744–0.823)
Social situations	3	6.30; 6; 0.390	0.714	0	0	0	0	0	0	1.637 (3%)	(−0.185, 0.208) [−4.020, 3.306]	(2.137, 2.294) [−0.874, 1.479]	0.877	0.763 (0.742–0.829)
Partner relationship	4	8.68; 8; 0.370	0.687	0	0	0	0	0	1	1.502 (2%)	(−0.641, 1.198) [−2.329, 3.831]	(2.401, 1.919) [−0.315, 0.763]	0.810	0.637 (0.587–0.703)
Positive Mood	5	11.22; 10; 0.340	0.894	0	0	0	0	0	0	1.535 (5%)	(−0.997, 1.264) [−6.881, 5.087]	(1.382, 2.547) [−0.387, 0.956]	0.909	0.771 (0.733–0.814)
Negative Mood	10	17.21; 20; 0.639	0.881	0	0	0	0	0	0	1.646 (3%)	(−1.020, 0.740) [−3.460, 4.461]	(2.266, 2.076) [−0.346, 1.073]	0.924	0.719 (0.636–0.788)
Parent concerns about the appearance of their child’s burn wounds/scars	4	4.73; 8; 0.786	0.830	0	0	0	0	0	0	1.476 (3%)	(−0.771, 0.597) [−3.090, 3.979]	(2.680, 2.692) [−0.363, 0.920]	0.937	0.854 (0.839–0.879)
Positive Growth	4	7.51; 8; 0.483	0.724	0	0	0	0	0	0	1.487 (5%)	(−0.335, 0.239) [−1.569, 2.288]	(0.729, 1.694) [−0.674, 1.375]	0.824	0.649 (0.564–0.720)

* Χ^2^ and F-statistic Baseline significance level was 0.01, which were simultaneously adjusted with Bonferroni correction for number of items; ** Criterion of unidimensionality assumed that highest eigenvalue for matrix of residual correlations should be <2.0; *** Criterion of unidimensionality acceptance is the proportion or lower bound of 95% confidence interval (95% CI LB), and is under 5%; **** Baseline significance level 0.05.

**Table 3:** Number (%) of participants to endorse each response category of the Parent Confidence Managing Burn Wound/Scar Treatments checklist.

Parent Confidence Managing Burn Wound/Scar Treatments *I Feel Confident Helping My Child with…*	Not at All		A Little Bit		A Bit		Quite a Bit		A Lot		N/A	
Scale/Item	N	%	N	%	N	%	N	%	N	%	N	%
1 …dressing/bandage changes	3	1.5	4	2.0	0	0.0	9	4.4	40	19.6	65	31.9
2 …creaming/massaging their burn scars	1	0.5	8	3.9	3	1.5	16	7.8	102	50.0	26	12.7
3 …washing and dressing/using the toilet	1	0.5	1	0.5	2	1.0	9	4.4	109	53.4	33	16.2
4 …physiotherapy exercises/stretching/putting on pressure garments/splinting	3	1.5	1	0.5	3	1.5	10	4.9	38	18.6	73	35.8
5 …taking their medication	1	0.5	0	0.0	2	1.0	5	2.5	41	20.1	68	33.3
6 …managing their discomfort (e.g., pain, itching)	1	0.5	6	2.9	6	2.9	18	8.8	54	26.5	51	25.0
7 …wound/scar treatments (e.g., dressing changes, creaming/massage, washing and dressing, physiotherapy exercises, pressure garments, splinting, taking medication)	2	1.0	3	1.5	4	2.0	17	8.3	75	36.8	46	22.5

## Data Availability

The data presented in this study are available in the article.
